# Analysis of Urban Built Environment Impacts on Outdoor Physical Activities—A Case Study in China

**DOI:** 10.3389/fpubh.2022.861456

**Published:** 2022-04-05

**Authors:** Bo Li, Qiuhong Liu, Tong Wang, He He, You Peng, Tao Feng

**Affiliations:** ^1^School of Architecture and Art, Central South University, Changsha, China; ^2^Management of the Built Environment Department, Architecture and the Built Environment Faculty, Delft University of Technology, Delft, Netherlands; ^3^Urban Planning and Transportation, Department of the Built Environment, Eindhoven University of Technology, Eindhoven, Netherlands; ^4^Graduate School of Advanced Science and Engineering, Hiroshima University, Higashi-Hiroshima, Japan

**Keywords:** public health, urban planning, GPS tracking, intensity and frequency, trajectory analysis, smart wearables

## Abstract

Outdoor physical activities can promote public health and they are largely influenced by the built environment in different urban settings. Understanding the association between outdoor physical activities and the built environment is important for promoting a high quality of life. Existing studies typically focus on one type of outdoor activity using interview-based small samples and are often lack of systematic understanding of the activities' intensity and frequency. In this study, we intend to gain deeper insight into how the built environment influences physical activities using the data extracted from individual's wearables and other open data sources for integrated analysis. Multi-linear regression with logarithm transformation is applied to perform the analysis using the data from Changsha, China. We found that built environment impacts on outdoor physical activities in Changsha are not always consistent with similar studies' results in other cities. The most effective measures to promote outdoor physical activities are the provision of good arterial and secondary road networks, community parks, among others in Changsha. The results shed light on future urban planning practices in terms of promoting public health.

## Introduction

Recent studies have shown that outdoor physical activities can improve people's physical and mental health by preventing chronic diseases, relieving anxiety, reducing depression, and other mental diseases rates (1). The urban built environment as a container in which such activities happen therefore affects residents' health (2, 3). The aging population in China has urged policymakers to design an urban environment that can promote outdoor physical activities. In China, the most favored outdoor physical activities are running and walking, with 71% and 51.5% of respondents stating that they had done so in 2020 and one of the reasons for choosing such activities is that these activities do not require extra equipment (4).

Regarding the research methods for urban built environment impacts on physical activities, some of the studies focus on the control of physical factors to analyze impacts (5). Furthermore, the subjective feeling of participants has been added to the controlling factors from the built environment (6). Such studies have proved that some of the built environment factors such as the accessibility of land, the design of slow traffic roads, and the visual level of the environmental landscape have an impact on outdoor physical activities (7). There have been also a large number of empirical research carried out using longitudinal study, cross-sectional study, and correlation analysis (8). Recently, the research perspectives are gradually diversified, and the research methods are evolving from original interviews, questionnaires, self, and expert evaluations to more objective technical methods, such as the combination of using remote sensing, geograhpic information systems (GIS) and global positioning systems (GPS) path tracking technologies (9).

Till now the commonly identified urban built environment factors' impacts on a combination of no-equipment outdoor physical activities which are generally applied in China (i.e., walking and running) (4, 10) is relatively less examined. Research especially from a purely built environment perspective is needed to help design urban built environments. Furthermore, local citizens' have their own preferences in China due to the size of each city. Therefore, it is desired to find a way to design a customized urban built environment based on local citizens' activities to stimulate outdoor walking and running activities and promote a healthy living lifestyle (11). Moreover, the current research mainly focuses on the impact analysis from one single perspective of physical activities like their intensity or frequency, in which intensity refers to the total length of each trajectory and frequency refers to the number of trajectories (12–15). However, to understand the outdoor physical activities' pattern better, different angles should be looked upon. As such, research is needed to systematically analyze urban built environment factors' impacts on walking and running from both frequency and intensity angles.

Furthermore, at present, most of the research is based on the research participants' home address, school, and workplace to establish a certain buffer for analysis, but less on the participant's motion trajectory (16–20). The latter, however, can provide a much richer source on identifying the correlations of the urban built environment and the outdoor physical activities (9, 21, 22). Besides, most of the existing research is based on means such as questionnaire surveys, on-the-spot or telephone interviews with a small amount of data, and incomplete questionnaires (5, 23). The development of smart wearables such as smartwatches provides us with a much richer data source to monitor even real-time human trajectories (24).

Open data practices such as open street maps (OSM) and satellite images offer opportunities to combine different urban scales with human trajectories. As such, the use of newly available data sources like trajectory data and open data to generate insights with fewer costs becomes feasible, using automatic scripts and tools from multiple platforms.

To summarize, the gap that we want to address in the current research is:

There is no systematic analysis of one city's urban built environment impacts on outdoor activities from different angles with the help of trajectory data and other open data sources.

Therefore, the research question raised in this study is:

*How most relevant urban built environment elements impact public outdoor walking and running activities, using data from wearables and other datasets, for a city like Changsha*.

The following part of this article is structured as follows: Background explains in more detail the literature review in relation to the case study area. Methods illustrates the methodology followed. Results and discussion explains the urban planning recommendations based on the analysis results. The Conclusion concludes the research.

## Background

Regarding impacting factors for outdoor activities, the current research can be divided into three categories. First, built environment factor impacts on physical activities are studied separately, such as the impacts of land-use mix (5, 6, 25–28), the connectivity of streets (29–31), perceived access to destinations (32–36), the environment quality of walking (9, 23, 37, 38), aesthetic feeling (39), and safety (16, 39, 40). Second, built environmental factors are combined to study impacts on the physical activities, such as the combination of park, greenbelt, and open space (21, 41, 42), the utilization of land-use mix and road density, the combination of public open space, and sports facilities (43, 44). Social factors such as living preferences and activity willingness of residents are added into the analysis together with the commonly combined built environment factors (45). Third, studies also discuss the impacts of the urban built environment on physical activity of different types (22, 46, 47) and by different age groups (10, 12, 48–53).

As shown, the existing studies are mainly focusing on one specific city and each city has its own characteristics and therefore, it is important to first understand the context so that we can identify the most relevant built environment factors for Changsha, in combination with the identified factors from literature.

### Context of the Research

Changsha is a city located in the Hunan province of the People's Republic of China. As one of the most urbanized cities in China, the aging population has urged the urban planners and decision-makers to provide facilities to support public health activities like outdoor walking and running activities. In China, the government applies 10 min walking distance as a general guideline for residential area planning. It means that within such buffers, it is desired that the public facilities can support outdoor activities. Therefore, in this study, we have applied an 800 m range to create grids for sampling and further analysis.

Changsha has also many universities which form an important destination for many people and therefore, in this research, we have specifically included accessibility to campus as an urban built environment factor to analyze.

### Urban Built Environment Factors and Activity Analysis Angels Identification

Based on the existing research results, the most identified factors which influence the urban walking and running activities in Changsha China are given in Table 1, together with the abbreviations (10, 11, 43, 54).

**Table 1:** Names and abbreviations of independent variables.

**Names**	**Abbreviations**
Land-use mix	LM
Residential land density	RLD
Building density	BD
Arterial road density	ARD
Secondary road density	SRD
Branch road density	BRD
Bus stop density	BSD
University campuses density	UCD
Comprehensive parks density	CPD
Community parks density	PD
Square density	SD
Market density	MD
Living service points density	LSD
Normalized difference vegetation index	NDVI

Research has shown that there is a positive correlation between the degree of mixed land use (LM) and the possibility of people engaging in active physical activity (55, 56). Specifically, Parra has studied the elderly as the research participant and found that if there are more types of land use in the area where the elderly are located, it is more possible to encourage the elderly to engage in walking activities and exercises to enhance their physical fitness (57). At the same time, the Density of residential land (RLD) also affects the occurrence of physical activities (58, 59). Considering the high urban land-use intensity in China, these factors are considered relevant for this case area.

The density of urban roads also has an impact on physical activities. As the carrier of residents' physical activities, urban roads are also channels connecting the functions and spaces of various parts of the city. The higher the connectivity in the neighborhood, the shorter the distance for residents to reach various target spaces, which is more conducive to increase relevant traffic physical activities, such as leisure physical activities like walking and running (20, 43). Research by Kelly et al. also shows that people who live in areas with high connectivity tend to have higher health levels (60). Moreover, different types of roads and their densities have varying degrees of impact on different types of physical activities (43, 61). Since Chinese cities like Changsha have numerous road networks, these typological factors on various levels are also incorporated in the study, namely, arterial road density (ARD), secondary road density (SRD), and branch road density (BRD).

Public facilities' density is also one of the main factors affecting the frequency and intensity of physical activities. A study found that more parks can effectively increase users' walking frequency and each additional piece of the facility will increase the probability of users' physical activity by 12% (62). In China, the densities of small community Parks (PD) and big comprehensive parks (CPD) are different and it is important to study how such facilities separately could affect outdoor physical activities. Other public facilities' densities such as square density (SD), living service points density (LSD), and market density (MD) are important for leisure physical activities, as well as bus stop density (BSD), and building density (BD) related to traffic physical activities (63). Generally speaking, higher accessibility means that residents can achieve the purpose of travel within a shorter distance, prompting residents to choose green travel methods such as walking, thereby encouraging residents to do physical activities outdoor. Studies have shown that being close to work and service places can promote walking, running, and other non-motorized vehicle travel, and increase traffic physical activities (64). The 16 universities located in Changsha are one of the most represented working places and destinations for residents. In addition, studies have shown that an open campus can effectively promote the physical activities of surrounding residents (65). As such, university campuses density (UCD) is included in the study.

Greenery, in general, has a general impact on outdoor physical activities. Areas with a high normalized vegetation index are more conducive to physical activity (66, 67). To generalize the factors, the normalized difference vegetation index (NDVI) is also applied in this research.

As mentioned in the introduction part, there is less research on combining the different angles of outdoor walking and running activities like frequency and intensity into one research, while it is important to combine these angles to fully understand such physical activities and design customized urban areas to promote these two activities accordingly. Therefore, this study has selected frequency and intensity as the dependent variable for walking and running activities (Table 2).

**Table 2:** Name and abbreviation of the dependent variable.

**Names**	**Abbreviations**
Walking activity frequency	WAF
Running activity frequency	RAF
Walking activity intensity	WAI
Running activity intensity	RAI

## Methods

In this section, the data collection and processing approaches are explained (Data collection and processing) together with the analysis models used (Multiple linear regression with logarithmic transformation).

### Data Collection and Processing

The built environment factors' data in the research area come from multiple sources, which mainly include urban land use type data, road network data, building vector data, urban point of interest (POI) data, and NDVI data. The urban land use type classification data comes from the master plan maps, which is vector data and is adjusted according to the most updated version. The road network data is processed and classified based on the data obtained from the OSM and the not completed parts are supplemented by the traffic status maps. The vector data of buildings is obtained through Gaode Map's open API (68) and calibrated according to the actual situations based on existing images. The city POI data (namely, universities, various parks, bus stops, squares, shopping malls, and life service points) are crawled through Gaode Map's API and corrected based on the real situations. The NDVI data are obtained from Landsat8 remote sensing images (69) through USGS (https://glovis.usgs.gov/). Landsat 8 TM image data are imported into ENVI, and radiometric calibration on the original data is performed. The calibrated data image is used to perform the atmospheric correction. After completing the radiometric calibration and atmospheric correction, NDVI calculation on the corrected TM image data is performed by the vegetation NDVI tool in the spectrum processing toolbar.

The outdoor walking and running physical activities' data and the trajectory data are obtained from smartwatch providers (Duorui wearable and application) and all the personal data have been deleted to protect the privacy and the users have all signed agreements to allow data gathering for research purposes. The research received a waiver of approval from the ethics committee, since it is not involving identifiable human subjects. The consent was given for data collection by all participants. To normalize the datasets and generate samples from the data obtained, the ArcGIS fishing net tool has been used to generate vector points in the study area. According to the actual situation of the urban built environment and the trajectory distribution in the study area, the sample points that fall on the water, non-built-up areas, and the surroundings are deleted. After this process, the sample size is obtained for further model development and analysis.

The dependent variables are calculated as follows: the frequency of physical activity is calculated by the number of trajectories in the sample using ArcMAP spatial connection tool. The intensity of walking and running activities is calculated by the total length of trajectory in the samples using ArcMAP clipping and spatial connection tools. With the help of ArcMAP intersection tool, the trajectory data within the research areas are screened out and the data are cleaned by deleting short motion time, short motion distance, and abnormal motion trajectories.

The independent variables in the models are calculated as follows:

- Land-use mix refers to the degree of diversification of urban land within a certain range. The entropy index model is used to determine the degree of land-use mixing.

(1) $$\begin{array}{r} LM=-\frac{\left(\sum_{i=1}^{N}P_{i}\;lnP_{i}\;\right)}{lnN} \end{array}$$

*where P*_*i*_ *is the proportion of the i*^*th*^ *type of land use in the current grid area, and N is the number of land use. The calculated land-use mixing degree value is a number from 0 to 1*.

- Residential land density refers to the ratio of the area of residential land within a certain range to the total area of the range.

(2) $$\begin{array}{r} RLD=\frac{RLA}{AREA} \end{array}$$

*where RLA is the area of residential land in the area (m*^2^*) and AREA is the total area (m*^2^*)*.

- Building density refers to the ratio of the area of the building base (BA) within a certain range to the total area of the range, reflecting the density of buildings.

(3) $$\begin{array}{r} BD=\frac{BA}{AREA} \end{array}$$

*where BA is the area of the building base (m*^2^*) and AREA is the total area (m*^2^*)*.

- Arterial road density refers to the ratio of the total length of the arterial road (ARL) within a certain range to the total area of the area.

(4) $$\begin{array}{r} ARD=\frac{ARL}{AREA} \end{array}$$

*where ARL is the total length of urban arterial roads in the area (m), and AREA is the total area (m*^2^*)*.

- Secondary road density refers to the ratio of the total length of the secondary road (SRL) within a certain range to the total area of the scope.

(5) $$\begin{array}{r} SRD=\frac{SRL}{AREA} \end{array}$$

*where SRL is the total length of secondary arterial roads in the region (m) and AREA is the total area (m*^2^*)*.

- Branch road density refers to the proportion of the total length of branch roads in a certain range to the total area of the range.

(6) $$\begin{array}{r} BRD=\frac{BRL}{AREA} \end{array}$$

*where BRL is the total length of urban branch roads in the area (m) and AREA is the total area (m*^2^*)*.

- University campuses density refers to the proportion of college campuses within a certain range to the total area.

(7) $$\begin{array}{r} UCD=\frac{UCA}{AREA} \end{array}$$

*where UCA is the area of college campuses in the region (m2) and AREA is the total area of the region (m*^2^*)*.

- Comprehensive parks density (refers to the proportion of big parks within a certain range to the total area.

(8) $$\begin{array}{r} CPD=\frac{CPA}{AREA} \end{array}$$

*where CPA is the area of comprehensive parks in the region (m2), and AREA is the total area (m*^2^*)*.

- Normalized difference vegetation index refers to the ratio of the average value of NDVI raster data pixel values to the number of pixels within a certain range, reflecting the vegetation coverage within a certain range.

(9) $$\begin{array}{r} NDVI=\frac{\sum_{i=1}^{N}NDVI_{i}}{N} \end{array}$$

*where NDVI*_*i*_ *is the DNVI value of a single pixel in the area, and N is the number of pixels in the area*.

- Community park density, bus stop density, square density, market density and living service points density refer to the number of facilities in a certain range. It is calculated based on POI data since these are all small facilities which make it not possible to calculate the area size. Therefore, nuclear density analysis in ArcGIS is first applied to generate a grid, and then the sample grid created is used to extract its grid density value.

### Multiple Linear Regression With Logarithmic Transformation

Multiple linear regression (MLR) is a statistical technique that employs several explanatory variables to predict the outcome of a dependent variable. The objective of MLR is to model the linear relationship between the explanatory variables and a dependent variable (70). More than one explanatory variable is involved in MLR which makes it an extension of ordinary least square regression with one explanatory variable. The formula equation of MLR is expressed as follow:

(10) $$\begin{array}{r} y=\beta_{0}+\beta_{1}x_{1}+\beta_{2}x_{2}+\cdots +\beta_{n}x_{n}+\varepsilon \end{array}$$

*where y denotes the dependent variable of observation, x*_*n*_ *denotes the nTH explanatory variable for the i*^*th*^ *observation, β_0_ is the intercept (constant), β_n_ is the slope coefficient for the nth explanatory variable, and ε is the error term (also known as residuals)*.

Multiple linear regression is a function that allows us to make predictions about dependent variables based on the information that is known about several independent variables. When applying MLR, there is a hypothetical linear relationship between the dependent variables and independent variables. The independent variables should not be highly correlated with each other. The observations of dependent variables must be selected independently and randomly from the sampled population. In addition, the residuals should be normally distributed with a mean of zero and variance σ.

In this study, since it cannot simply take the linear relationships between dependent and independent variables for granted, we used MLR-LT of the dependent variable to avoid the violation of the non-linear relationship which may potentially exist between the dependent and independent variables. The logarithmic transformation is also convenient means of transforming a highly skewed distribution of variables into a normally shaped bell curve. In addition, taking the logarithm of the dependent variable can effectively vary the unit change of independent variables to the percent change. This logarithm transformation is especially important when it comes to using a large dataset. The MLR-LT is expressed as:

(11) $$\begin{array}{r} ln\;y=\beta_{0}+\beta_{1}x_{1}+\beta_{2}x_{2}+\cdots +\beta_{n}x_{n}+\varepsilon \end{array}$$

The *R*^2^ is used as a metric to measure how much the variation in the dependent variable can be statistically explained by the variation in the independent variables. The value of *R*^2^ is between 0 and 1, where 0 means the outcome of the dependent variable cannot be predicted by any of the independent variables, and 1 indicates the outcome of the dependent variable can be perfectly predicted by the independent variables without any errors. In estimation, the slope coefficients are valid while holding all the other variables constant. The *R*^2^ is monotonous increasing with the number of variables, which leads to the alternative of adjusted *R*^2^ ($R_{adjusted}^{2}$). The $R_{adjusted}^{2}$ penalizes the statistic outcomes caused by extra variables.

In the regression analysis, many independent variables may affect the frequency and intensity of physical activities; the independent variables are eliminated one by one in the correlation analysis using SPSS software. Then the model's collinearity is checked. The independent variable factors of the urban built environment with correlations are obtained. As such, the influence equations of walking frequency, running frequency, walking intensity, and running intensity are obtained.

## Results and Discussion

This section illustrates how the developed methodology is applied in the Changsha region in China. The first part explains the area and the data sources in more detail, followed by the spatial and temporal distribution characteristics of the collected trajectories for further analysis. To combine the urban built environment factors with the trajectories of outdoor physical activities, a spatial processing process is performed to generate the sample trajectories in the urban built environment (Preliminary analysis results). MLR models with logarithmic transformation are generated using the sample trajectories (Result of MLR-LT). The following part of this section illustrates the analysis results and the indications on urban built environment planning practice (Discussion).

### Preliminary Analysis Results

**Figure 1:**
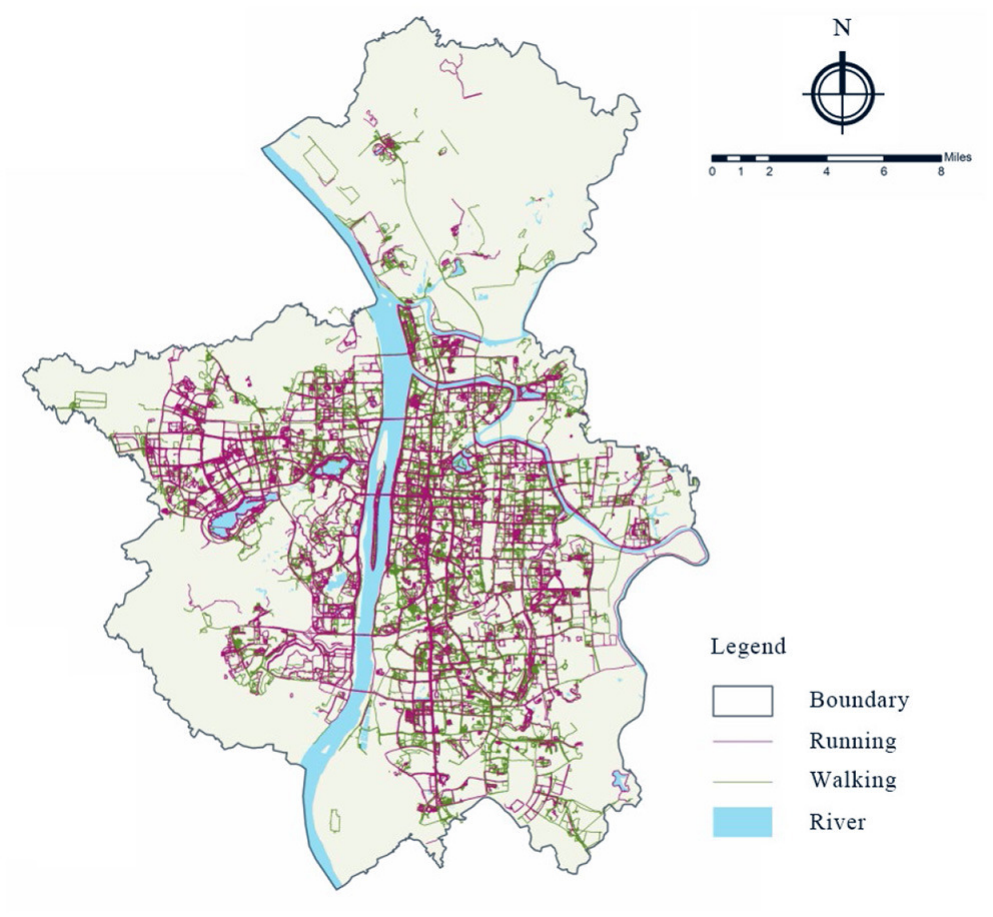
Distribution of slow motion physical activity trajectories (pink for running, green for walking).

Data on walking and running activities were collected in Changsha from the year 2016 to the year 2018. Based on the 35,019 effective samples of trajectories, we can not only obtain the information of physical activity types but also other information such as spatial locations, starting time, duration, and distance. In particular, 10,773 effective walking data and 5,879 effective running data are obtained, as shown in Figure 1.

The data used for characterizing the built environment are getting from the master plan maps of Changsha (2003–2020), OSM and the traffic status maps of Changsha (2017–2035), Gaode Map API (68), and Landsat8 remote sensing images (69). Through the preliminary analysis of the motion data, most of the trajectories are distributed in the main urban area of Changsha city, while the trajectories in other areas are less scattered. Therefore, the main urban areas of Changsha, including Tianxin district, Yuhua district, Kaifu district, Furong district, and Yuelu district (excluding Pingtang street, Hanpu street, Yuchangping town, and Lianhua town) are selected as the study areas. The research areas cover 71 streets with a total area of 817 square km. The data is further cleaned by deleting trajectories with position offset, short motion time (<3 min), short motion distance (<0.4 km for walking and <0.185 km for running), and abnormal motion factors like speed <1 km/h. These thresholds are set by the smart wearables' providers.

**Figure 2:**
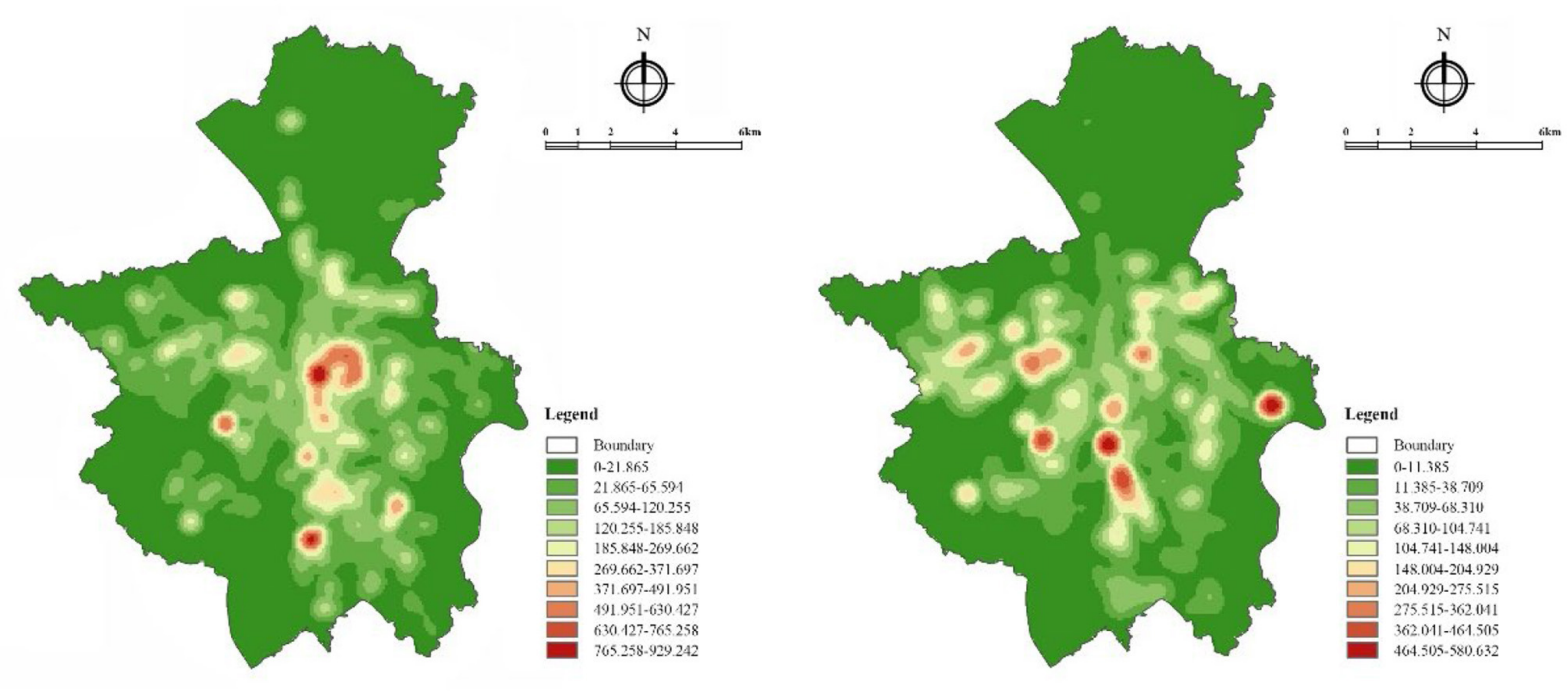
Trajectory density distribution of walking (left) and running (right) activity.

Some differences in the spatial and temporal distribution have been found between walking and running. From the perspective of time distribution, people mostly go on physical activities in the morning (5:00–8:59) and evening (18:00–20:59), and the average activity time of walking is longer than that of running, while the average activity distance and average activity speed are lower than that of running. From the perspective of spatial distribution, walking and running activities are distributed in the areas with relatively good urban construction conditions, but the scope of walking is larger than that of running. Compared with walking activities, running activities are less concentrated (shown in Figure 2).

**Figure 3:**
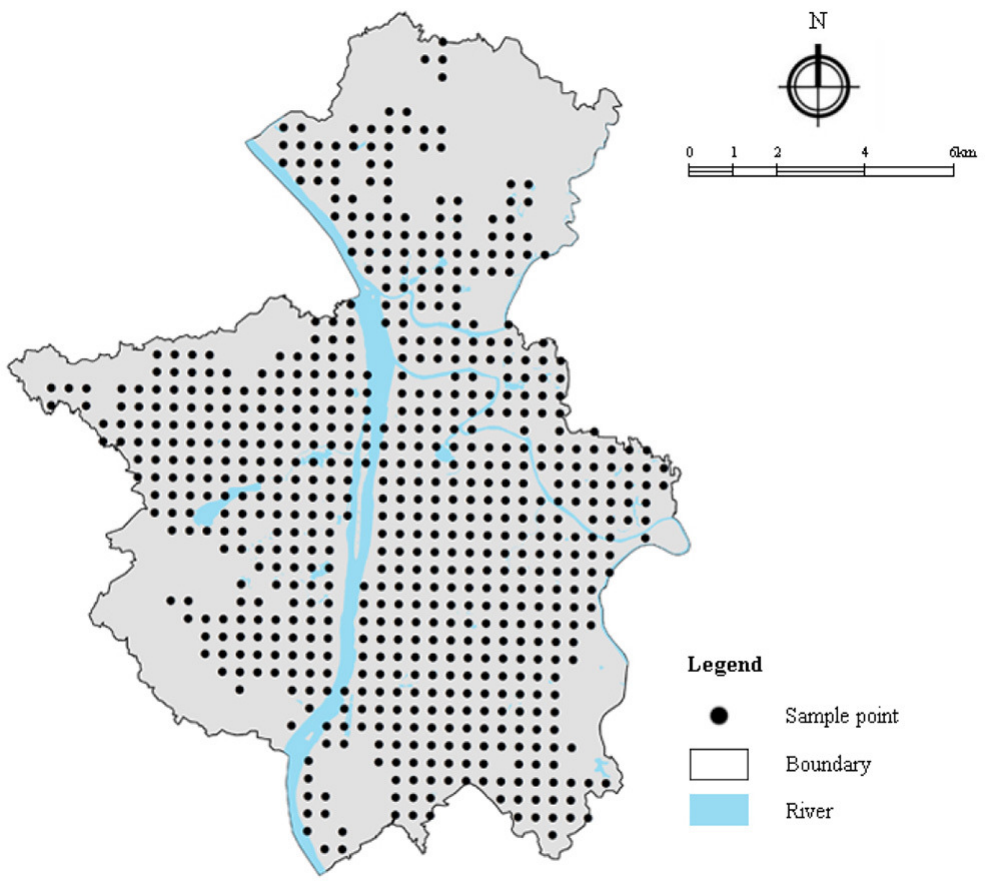
Sample points in the case study area.

Vector points with a distance of 800 m in the study area are generated to normalize the datasets. After deleting sample points that fall on the water, non-built-up areas, and the surroundings, there are 735 samples left for further analysis (Figure 3).

### Result of MLR-LT

The *R*^2^ of each MLR-LT is shown in Table 3, which gives information on how well the regression predictions approximates the sampling data. Accordingly, with *R*^2^ and $R_{adjusted}^{2}$ exceeding 0.6, the models regarding WAF and WAI fit the data well. Whereas, the *R*^2^ and $R_{adjusted}^{2}$ of models regarding RAF and RAI indicate that the model of RAF accounts for around 40% of the variance between observed dependent variables and predicted dependent variables, while the model of RAI accounts for around 50%. Regardless of the *R*^2^, the significant coefficients still represent the mean-variance in the dependent variables for one unit of change in the independent variable while holding other independent variables in the model constant.

**Table 3:** The goodness-to-fit of all the estimates of multiple linear regression with logarithmic transformation (MLR-LT).

	**WAF**	**RAF**	**WAI**	**RAI**
**R** ^**2**^	0.620	0.422	0.632	0.535
${\text{R}}_{\text{adjusted}}^{2}$	0.612	0.409	0.625	0.525

The estimated results regarding the frequency of walking and running are shown in Table 4.

**Table 4:** The results of MLR-LT regarding WAF and RAF.

**Variable**	**WAF**		**RAF**	
	**β**	***P*-value**	**β**	***P*-value**
LM	0.064	0.007	0.032	0.299
RLD	0.220	0.000	0.148	0.001
BD	0.049	0.300	0.052	0.397
PD	0.163	0.000	0.229	0.000
UCD	0.140	0.000	0.264	0.000
ARD	0.209	0.000	0.132	0.003
SRD	0.101	0.004	0.234	0.000
BRD	0.116	0.016	0.049	0.429
LSD	−0.059	0.338	−0.160	0.045
CPD	0.083	0.009	0.028	0.486
BSD	0.127	0.022	0.061	0.385
MD	0.042	0.338	0.102	0.071
SD	0.103	0.009	0.120	0.020
NDVI	0.085	0.001	0.013	0.691

The walking and running frequency are both significantly influenced by the residential land density (RLD), community parks density (PD), university campus density (UCD), square density (SD), arterial road density (ARD) and secondary road density (SRD) in a positive way. It is easy to understand the effect of RLD since the location of the home is normally the start point of outdoor activities. The spaces and facilities in the park, university campus, and square are conducive to physical activities. The arterial and secondary roads are also providing routes for walking and running, especially in dense urban environments.

The land use mix (LM), normalized difference vegetation index (NDVI), branch road density (BRD), bus stop density (BSD) and community park density (PD) are proportional to the frequency of walking, whereas their effects on running frequency are not significant. This finding distinguishes the different needs of spatial settings and infrastructures across walking and running activities. The land-use mix level has significant positive impacts on walking frequency, which could be explained by the multiple intentions of people for activities, like grocery shopping, meeting friends, visiting green spaces, etc. The increase of NDVI results in more greenery coverages, which to some extent attracts light outdoor activities, resting, walking, among others. The activity that happens close to vegetation, to some extent, implies people's preference for greenery when walking in outdoor urban environments. However, NDVI is not significantly related to the frequency of running. In real-world contexts of the study locations, the mixed-use branch roads with sidewalks are leading to the nearby neighborhoods, shops, restaurants, and other destinations. It is reasonable to assume that, during walking, residents may carry out other activities simultaneously as above mentioned. The BSD and PD are increased in the areas of more residential neighborhoods. Therefore, the positive effect of the BRDs, BSD, and PD on walking makes sense. On the other hand, the runners must start and end the running in their residential neighborhoods where many branch roads, bus stops, and community parks are located, but they may avoid the branch roads, bus stops even community parks in other neighborhoods, since they are not the right places for running.

The living service points density (LSD) only significantly impacts the frequency of running. The increase of LSD results in less frequency of running. For the sake of efficiency and running conditions, the runners are inclined to avoid the areas with living service points on purpose. Thus, less frequent running trajectories come out in a place with a high LSD.

Concerning the intensity of walking and running activities, the results of the estimation are shown in Table 5. The positive effects regarding residential land density (RLD), community park density (PD), university campus density (UCD), square density (SD), comprehensive park density (CPD), arterial road density (ARD) and secondary road density (SRD) on both walking and running intensities are significant, which is similar with the effects of these variables on the frequency of walking and running activities. The provision of spaces and facilities is still the reason that the higher density of these places and infrastructures are favorable to the increase of the intensity of walking and running activities. The normalized difference vegetation index (NDVI) also has a positive influence on both walking and running intensities.

**Table 5:** The results of MLR-LT regarding WAI and RAI.

**Variable**	**WAI**		**RAI**	
	**β**	***P*-value**	**β**	***P*-value**
LM	0.051	0.028	0.021	0.453
RLD	0.217	0.000	0.151	0.000
BD	0.021	0.644	0.070	0.204
PD	0.165	0.000	0.196	0.000
UCD	0.153	0.000	0.292	0.000
ARD	0.248	0.000	0.195	0.000
SRD	0.115	0.001	0.319	0.000
BRD	0.115	0.016	−0.023	0.674
LSD	−0.089	0.140	−0.162	0.024
CPD	0.130	0.000	0.131	0.000
BSD	0.106	0.052	0.026	0.682
MD	0.048	0.258	0.019	0.708
SD	0.103	0.008	0.185	0.000
NDVI	0.144	0.000	0.127	0.000

Land-use mix (LM), branch road density (BRD) and bus stop density (BSD) are proportion to the walking intensity, whereas they are not significantly related to the intensity of running. Compared with running, walking is more relaxed and less constrained by the tempo-spatial settings. In some cases, the walking activity is combined with leisure, shopping, social activities in pedestrian business areas, where many brand roads and bus stops are located nearby.

The living service points density (LSD) negatively influences running intensity which is similar to its effect on running frequency. The market density (MD) has no direct significant impact on both walking and running intensities.

Multicollinearity will decrease the statistical significance of the regression model. As the diagnosis for multicollinearity, the variance inflation factor (VIF) of MLR-LT is given in Table 6. Accordingly, the VIFs indicate no multicollinearity for all independent variables (all <10).

**Table 6:** The variance inflation factor of MLR-LT.

**Variable**	**WAF**	**RAF**	**WAI**	**RAI**
LM	1.042	1.030	1.042	1.030
RLD	2.075	1.954	2.075	1.954
BD	4.190	4.102	4.190	4.102
PD	1.075	1.098	1.075	1.098
UCD	1.084	1.088	1.084	1.088
ARD	2.209	2.045	2.209	2.045
SRD	2.244	2.018	2.244	2.018
BRD	4.354	4.194	4.354	4.194
LSD	7.135	6.887	7.135	6.887
CPD	1.884	1.726	1.884	1.726
BSD	5.767	5.348	5.767	5.348
MD	3.542	3.467	3.542	3.467
SD	2.950	2.890	2.950	2.890
NDVI	1.194	1.214	1.194	1.214

### Discussion

The increase of land-use mix (LM) will promote the frequency and intensity of walking while there is no correlation found of land use mix with the frequency and intensity of running activity. This is probably because the higher land-use mix means more abundant destinations and urban functions, which will increase the willingness and intensity of residents to carry out walking activities that can help them achieve several goals like grocery shopping, exercising, and picking up packages. For urban planners, it is important to understand the local communities' preference for outdoor activities to design a balanced land-use mix and prevent unnecessary traffic flow to promote walking activities.

The residential land density (RLD) has positive impacts on both running and walking activities regarding intensity and frequency. This could be explained by that residents all need to start and come back to their residence. However, we can see that RLD has a higher impact on walking activities than on running activities which is probably because runners favor places with more open areas rather than the high residential building density areas.

Interestingly, building density (BD) and market density (MD) have no correlation with both running and walking activities, which is in contrast with the literature.

Community parks and squares play also a very important role. The results show that PD and SD have a high positive correlation with the frequency and intensity of walking and running activities, and the positive impacts are higher for running than that for walking. Compared with the other types of leisure places like a comprehensive park, the community parks and squares are smaller in overall area and less perfect in facilities and landscape, but they are normally more accessible and relatively easy to develop, which makes up for the lack of service scope of the comprehensive parks to a certain extent. In contrary to the PD and SD, the CPD has a fewer impact on walking activities and no direct influence on running frequency, but it does have positive impacts on running intensity. It could be explained that comprehensive parks are not that many, and it is not common for people to run that far. Therefore, community parks and squares can effectively promote the intensity of residents' activities in a certain range. So, community parks and squares should be further promoted in densely populated cities urban planning practice.

University campus density is with even higher correlations to walking and running activities, compared with community parks and the impacts of UCD on running activities is much higher than that on walking activities. This indicates that the campus space carries an important role in promoting physical activities, especially for running. Potential guidelines to use the open campus as a running activity base could be developed, particularly for cities like Changsha which has many universities.

Different types and levels of urban road density affect the frequency and intensity of physical activities on different levels. The urban road factors selected in this paper include arterial road density (ARD), secondary road density “SRD” and branch road density (BRD), and the results show that the urban ARD has a big positive correlation with the frequency and intensity of physical activity, and it has more impacts on walking activities than on running activities. From the regression results, the influence of urban SRD is still positive, but SRD promotes running activities more than promoting walking activities. However, the BRD only has a limited amount of positive impact on walking activities while no direct correlation with running activities. It could be argued that based on the results, context-specific road network designs can be improved. For example, the increase of SRD will significantly promote the frequency and intensity of running activities and the increase of ARD will significantly promote walking activities. For areas with more aging populations, the road network could be with more branch roads to promote walking activities.

Living service points density has no correlation with walking activity but a bit negative influence on running activities, which could be explained that the living service points we have referred to here are all facilities running during daytime while the walking activities are mainly happening not during the daytime and running activities are not prone to stops.

The normalized difference vegetation index (NDVI) mainly affects walking activities. The results show that walking is more likely to be carried out in the urban environment with greenery, while running may have lower requirements for landscape greening than walking, and it is more likely to choose the path with connectivity and comfort and NDVI has no direct correlation with running frequency but positive impact on running intensity. Therefore, for urban planners, general greenery should be provided to promote walking activities and if combined with parks and squares, it could potentially stimulate running intensities.

## Conclusion

The highly populated and urbanized cities in China ask for high-quality built environments that can facilitate and stimulate outdoor physical activities to benefit social cohesion and public health. In the literature, we have found quite some factors that can influence outdoor physical activities. However, three main limitations still remain. First, the findings of previous studies are mainly case-based without consideration of environmental conditions, which need to be fully understood while designing a generic approach to generate insights. Furthermore, the previous studies have not analyzed physical activities from angles of frequency and intensity. Third, the size of sample data is limited in the existing literature. This research proposes a conceptual framework with the comprehensive influences of urban built environments for identifying commonly found urban built environment factors that influence people's walking and running frequency and intensity for a case city.

This study has, therefore, contributed to: (1) developing a systematic analysis approach for the relationships between the urban built environment factors and outdoor physical activities for a city; (2) identifying effective and significant influential factors based on the city context; (3) presenting the method of collecting and processing data from smart wearables and OSM, Gaode and remote sensing data to analyze outdoor physical activities trajectories and patterns in the urban built environment; (4) addressing the process and results of GIS spatial analysis to obtain physical activities' intensity and frequency and cleaning trajectory data to generate realistic samples; (5) developing multiple models to analyze the impacts of multiple urban built environment factors on two types of outdoor activities, from both frequency and intensity angles; and (6) generating urban planning insights based on the frequency and intensity analysis of the most two favored outdoor activities for the case study region.

This research collects data generated from smart wearables and open geographical datasets like OSM, Gaode Maps, and remote sensing images. The approach is feasible for investigations on urban outdoor physical activities in other regions. Moreover, the findings of this study have implications for related urban policymaking in Changsha and other cities in China with similar environmental and socio-cultural contexts. To be more specific, such findings can help decision-makers in their planning activities as a starting point for discussing with citizens in the pursuit of a better urban built environment which helps to promote public health. Possible starting points could be providing more community parks, maintaining or improving road networks, designing open campuses, etc.

More angles from physical activities like starting time and duration are expected in the future studies. On one hand, analysis of the relationships between built environment characteristics and other outdoor physical activities, such as cycling, dancing, etc., can be followed using the same approach. In addition, the detailed analysis can be performed regarding the difference between recreational walking and transport walking (71). On the other hand, incorporating social factors, and the subjects' demographic information and health-related data may predict citizens' preferences in a certain region and therefore improve the neighboring facilities to promote physical activities, design a flexible and adaptive urban environment that can fit different purposes and help in designing a more customized urban environments for different users. A guideline for integrating different data sources is expected to be developed, together with an automatic approach for the data collection and processing tasks in potential cases.

## Data Availability Statement

The data analyzed in this study is subject to the following licenses/restrictions: Some data is not publically available, and follows commercial license. Requests to access these datasets should be directed to BL libo0910@csu.edu.cn.

## Ethics Statement

All the personal data have been deleted to protect the privacy and the users have all signed agreements to allow data gathering for research purposes. The research received a waiver of approval since it is not involving identifiable human subjects. Written informed consent was given for data collection by all participants.

## Author Contributions

BL, QL, and HH: conceptualization and methodology. QL: software, data curation, and visualization. QL, YP, TF, and TW: formal analysis. BL, HH, and TW: resources. QL and TW: writing—original draft preparation. TW, YP, and TF: writing—review and editing. BL, HH, YP, and TW: supervision. All authors have read and agreed to the published version of the manuscript.

## Funding

The APC is funded by the Delft University of Technology.

## Conflict of Interest

The authors declare that the research was conducted in the absence of any commercial or financial relationships that could be construed as a potential conflict of interest.

## Publisher's Note

All claims expressed in this article are solely those of the authors and do not necessarily represent those of their affiliated organizations, or those of the publisher, the editors and the reviewers. Any product that may be evaluated in this article, or claim that may be made by its manufacturer, is not guaranteed or endorsed by the publisher.
